# Evaluation of Morpho-Physiological Traits Adjustment of *Prosopis tamarugo* Under Long-Term Groundwater Depletion in the Hyper-Arid Atacama Desert

**DOI:** 10.3389/fpls.2018.00453

**Published:** 2018-04-09

**Authors:** Marco Garrido, Herman Silva, Nicolás Franck, Jorge Arenas, Edmundo Acevedo

**Affiliations:** ^1^Soil-Plant-Water Relations Laboratory, Agricultural Production Department, Faculty of Agronomical Sciences, Universidad de Chile, Santiago, Chile; ^2^Faculty of Natural Renewable Resources, Desert Agriculture, Universidad Arturo Prat, Iquique, Chile

**Keywords:** leaf isotopic composition, hydraulic lift, phreatophyte, stomatal behavior, water table depth

## Abstract

Water extraction from the underground aquifers of the Pampa del Tamarugal (Atacama Desert, Chile) reduced the growing area of *Prosopis tamarugo*, a strict phreatic species endemic to northern Chile. The objective of this work was to evaluate the effect of various architectural and morpho-physiological traits adjustment of *P. tamarugo* subjected to three groundwater depletion intervals (GWDr): <1 m (control), 1–4 m and 6–9 m. The traits were evaluated at three levels, plant [height, trunk cross-section area, leaf fraction (*f*GCC), and crown size], organ [length of internodes, leaf mass per unit area (LMA), leaflet mass and area], and tissue level [wood density (WD), leaf ^13^C, ^18^O isotope composition (δ), and intrinsic water use efficiency (iWUE)]. In addition, soil water content (VWC) to 1.3 m soil depth, pre-dawn and midday water potential difference (ΔΨ), and stomatal conductance (g_s_) were evaluated. At the deeper GWDr, *P. tamarugo* experienced significant growth restriction and reduced *f*GCC, the remaining canopy had a significantly higher LMA associated with smaller leaflets. No differences in internode length and WD were observed. Values for δ^13^C and δ^18^O indicated that as GWDr increased, iWUE increased as a result of partial stomata closure with no significant effect on net assimilation over time. The morpho-physiological changes experienced by *P. tamarugo* allowed it to acclimate and survive in a condition of groundwater depletion, keeping a functional but diminished canopy. These adjustments allowed maintenance of a relatively high g_s_; ΔΨ was not different among GWDrs despite smaller VWC at greater GWDr. Although current conservation initiatives of this species are promising, forest deterioration is expected continue as groundwater depth increases.

## Introduction

Climate change and human intervention have contributed to the reduction of productivity and accelerated death rate of forests in diverse ecosystems (Allen et al., 2010; Williams et al., 2013). This is the case of the Pampa del Tamarugal, a hyper-arid ecosystem in northern Chile, having groundwater as the only significant water source, given the near-absolute absence of rainfall (Pliscoff and Leubert, 2006). The Pampa del Tamarugal is a central plateau at about 1,000 m altitude bordered by the Andes in the east and a Coast range in the west; the groundwater flows from the high Andes and forms a subterranean lake in the central plateau. In this environment grows *Prosopis tamarugo*, an endemic legume tree, highly adapted to the extreme temperature and radiation of the Pampa del Tamarugal (Lehner et al., 2001; Chávez et al., 2013). *P. tamarugo* is an evergreen species, which grows throughout the year with a maximum in the spring-summer period, and a partial defoliation in winter, when the minimum temperatures are below zero °C (Sudzuki, 1969). *P. tamarugo* has a pivotal root system that access the groundwater as well as system of sub-surface lateral roots that grow in the highly saline upper soil, exerting hydraulic lifting (Aravena and Acevedo, 1985).

The groundwater extraction for urban, agricultural, and mining uses in the Pampa del Tamarugal has generated a negative water balance since 1988 (Rojas et al., 2013). The water depletion of the water table is decreasing the growing area of this species, presently been classified as endangered. The *P. tamarugo* forest decline in the Pintados and Bellavista salt flats (northern area of the Pampa del Tamarugal National Reserve) has been quantified by Chávez et al. (2016), who indicated that, in 2011 out of a total of 73,000 trees, 42% had less than 0.5 green coverage fraction (range from 0 to1). The study showed that *P. tamarugo* survival is at risk, and it is therefore necessary to enforce conservation measures. In desert ecosystems such as Pampa del Tamarugal, the decline and loss of ecosystem services, such as atmospheric carbon capture and hydrological regulation, are particularly significant given the scarcity of vegetation (Bidak et al., 2015). It follows that understanding the tolerance and acclimation mechanisms that *P. tamarugo* has to cope with water stress would help to predict its chance of survival as well as to design management alternatives to mitigate harmful anthropogenic and climate change effects.

The physiological stress intensity experienced by a tree is determined by a set of morpho-physiological and structural features associated with water absorption, conduction, and loss (Cruiziat et al., 2002; Addington et al., 2006). Facing a water stress, initially the plants are reported to reduce its stomatal conductance with gas exchange consequences (Eamus et al., 2013), what has been studied under natural conditions using stable carbon isotopes in organic matter (Zolfaghar et al., 2017). In the medium-to-long term, the trees make anatomical-structural modifications leading at maintaining either their water transport efficiency (water transport capacity) and/or safety (increased cavitation resistance) of their hydraulic systems, existing a trade-off between these hydraulic strategies (Tyree et al., 1994; Maherali et al., 2004; Sperry et al., 2008). Addington et al. (2006) found that *Pinus palustris* in a xeric habitat were shorter in stature, with lower sapwood-to-leaf area ratio, and a higher root-to-leaf area ratio than trees in a mesic habitat. A similar trend was observed in facultative phreatophytes (*Banksia attenuata* and *B. menziesii*) growing at a more xeric dune crest site having a xylem more resistant to embolism than trees growing at the wetter bottom slope (Canham et al., 2009).

It is likely that *P. tamarugo*, a species adapted to extreme aridity, would opt to deploy a strategy of hydraulic safety under water stress, investing in producing smaller-diameter xylem vessels (Sperry and Saliendra, 1994; Mainzer et al., 2009) associated with a denser wood, since narrow vessels are immersed in relatively dense wood matrices with more fibers in the xylem tissue (Hacke et al., 2001; Bucci et al., 2004; Fortunel et al., 2014). It should also have a greater investment in root growth (De Micco and Aronne, 2012) and conductive tissue, as well as lower investment in above-ground biomass (Gotsch et al., 2010; Gehring et al., 2016). Leaf area should be reduced via defoliation (Rood et al., 2000; Zolfaghar et al., 2017), and leaf mass per unit area should be increased, thereby increasing wilting resistance (Bucci et al., 2004; Poorter et al., 2009; Shadwell and February, 2017). The deployment of these adjustments should keep a relatively high rate of transpiration per unit leaf area, maintaining gas exchange, however, exposure to lower water potentials could lead to generalized cavitation.

Recent studies show that in conditions of severe groundwater depletion (water table decrease > 6 m), *P. tamarugo* decreases its xylem water potentials and maintain a relatively high stomatal conductance measured with porometry, along with a ^18^O isotopic enrichment in the leaf tissue (Ortiz, 2010) associated with partial stomata closure (Farquhar et al., 2007). In addition, trees experiment a partial defoliation (Ortiz, 2010; Chávez et al., 2013), and a lower investment in radial growth of lateral branches (Decuyper et al., 2016) as well as twig length (Squella, 2013).

The present work was done in a native relict forest of *P. tamarugo* located in the Salar de Llamara, Atacama Desert, Northern Chile. This formation is a thorny tropical forest, composed mainly of *P. tamarugo* and *Caesalpinia aphylla*. Groundwater extraction for mining has occurred from the end of 2005, generating a significant water depletion. Field observations reveal a decline in *P. tamarugo* health in the area (Garrido et al., 2016), but no deaths have been recorded. Our working hypothesis is that under water stress due to the decrease of the water table, *P. tamarugo* adjusts morphological and structural traits at various levels (canopy, organs, and tissues) leading to maintain gas exchange and hydraulic safety at canopy level. Our objective was to evaluate the effect of the adjustment of various morpho-physiological and structural traits on the water status of *P. tamarugo* after 10 years of groundwater depletion.

## Materials and methods

### Experiment and environmental condition

**Figure 1:**
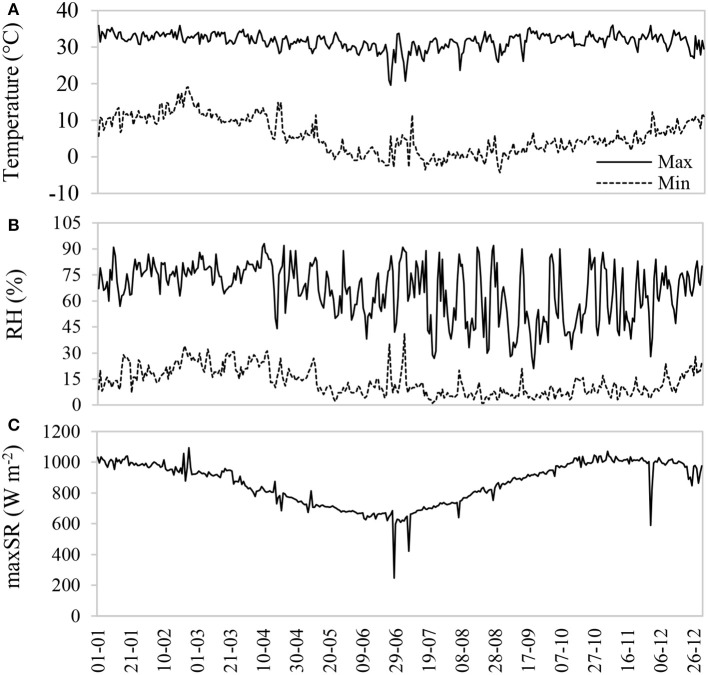
Maximum and minimum air temperature **(A)**, relative humidity **(B)**, and maximum daily solar radiation **(C)** during 2016 at the Canchones Experimental Station, Pozo al Monte Comuna, Tamarugal Province (19K 444490.5 m E; 7739128.5 m S).

The study was conducted in the Salar de Llamara (19K 434222 m E, 7658495 m S), a tropical hyper-desert bioclimate (Pliscoff and Leubert, 2006) located in the southern extreme of the Pampa del Tamarugal National Reserve, Northern Chile. This place is a native arborescent scrubland of very low plant density, consisting primarily of *P. tamarugo* (<10% coverage, with 0.83 trees ha^−1^) and *Caesalpinia aphylla* (<25% coverage). The zone is characterized by high thermal oscillation, low relative humidity, and high incident radiation (Figure 1). For more information about the study site, see Calderón et al. (2015). During 2016, the rainfall was 0.6 mm, and between 2011 and 2015 the accumulated rainfall was 6 mm. The maximum temperature at the sampling dates was 33.1 ± 1.3°C in January, and 30.7 ± 2.3°C in August. The minimum temperature had a greater fluctuation over the year, with monthly averages of 10.4 ± 1.9°C and 0.4 ± 2.0°C for January and August, respectively. A high frequency of below zero temperatures was observed during the winter. The maximum and minimum relative humidity were higher in the summer than in the winter, and the maximum daily incident solar radiation fluctuated between 1000 ± 21.9 and 787 ± 47.1 W m^−2^ between January and August 2016.

Groundwater extraction for mining at the Salar de Llamara started at the end of 2005 (Table 1), causing a groundwater depletion (GWD) gradient around the extraction point (Ortiz, 2010; Garrido et al., 2016). Sixteen *P. tamarugo* trees were selected along a 4.9 km north-south linear transect, starting at the water extraction point. Piezometric wells along the transect allowed determining the current and historic phreatic depths (Table 1; www.odea.cl). With this information, three groundwater depletion ranges (GWDrs) were defined: (i) <1 m (five trees subjected to a GWDr of less than 1 m), (ii) 1–4 m (four trees in a GWDr between 1 and 4 m), and (iii) 6–9 m (seven trees in a GWDr between 6 and 9 m). Well PO-01, located 3.2 km from the extraction site, had a GWD of 1.2 m in August 2016 (Table 1). It was assumed that all trees located beyond that well along the transect were subject to a GWDr lower than 1 m.

**Table 1:** Geographic coordinates and water table depths measured at eight observation wells in the Salar de Llamara, Pampa del Tamarugal, Chile.

**Observation well**	**DAP (m)**	**mE and mS Coordinates**		**Altitude (m)**	**Groundwater depth/depletion (m)**		
					December 2005^*^	**January 2016**	**August 2016**
P00	0	434195	7659433	754	3.3	39.3/36.0	38.1/34.8
PO-7a	684	434410	7658870	753	4.0	15.8/11.8	14.7/10.7
PO-6a	716	434400	7658748	748	3.9	14.9/11.0	15.0/11.1
PO-5	886	434538	7658712	751	4.2	13.0/8.8	13.4/9.2
PO-3	983	434580	7658533	750	4.4	11.1/6.7	11.6/7.2
PO-4	1631	434628	7657951	755	5.1	10.3/5.2	10.6/5.5
PO-8	2238	434430	7657228	746	4.6	7.3/2.7	7.5/2.9
PO-1	3192	434422	7656606	747	5.9	7.0/1.1	7.1/1.2

*Well P00 is located at the point of water extraction, therefore it shows the dynamic level. The distance from each well to the water extraction point (DAP) is indicated*.

* *Water extraction operations started in December 2005*.

Measurements were made in January and August 2016, representing the summer (more active vegetative growth period) and winter seasons (lower vegetative growth and partial defoliation), respectively.

### Water content and soil chemical properties

Soil samples were taken between 0 and 130 cm depth soil profiles at regular 20 cm intervals under the canopy of four *P. tamarugo* trees (two trees in GWDr <1 m, and two in the 6–9 m GWDr) in January and August. Samples were put in aluminum capsules, weighed to determine wet weight, and dried at 105°C to determine the weight of dry soil, and the gravimetric water content was calculated. The bulk density at each depth was measured independently to express the water content on a volumetric basis (θ; cm^3^ cm^−3^). Approximately 1 Kg soil was sampled in January at 50 and 100 cm depth. These soil samples were sent to the soil chemistry laboratory of the regional INIA Center (La Platina) for pH, electrical conductivity (EC, dS m^−1^), organic matter content (OM; %), total available nitrogen (N; mg Kg^−1^), soluble potassium (K; mg Kg^−1^), and Olsen phosphorus (P; mg Kg^−1^) analyses.

### Tree water relations

Xylem water potential (twigΨ; MPa) and stomatal conductance (g_s_; mmol m^−2^ s^−1^) were measured during summer and winter at a height of 1.5 m in the side facing northeast of each tree in the <1 and 6–9 m GWDrs (*n* = 5 and *n* = 7, respectively). Xylem water potentials were measured at pre-dawn (twigΨ_pd_; around 6:00 in summer and around 7:00 in winter; dawn in the area is 06:44 in January and 07:50 in August), and midday (twigΨ_md_; between 13:30 and 15:00). The measurements were made following a standard methodology (Scholander et al., 1965) with a pressure chamber (1505D EXP model; PMS Instrument Company). Four twigs per tree were covered with aluminum and plastic bags during the night for twigΨ_pd_ measurement, and four other twigs were covered during 2 h for twigΨ_md_ measurement. The g_s_ was measured between 13:30 and 15:00 (at the same time and in the same trees where twigΨ_md_ was measured), with an SC-1 Steady State Diffusion Leaf Porometer (Decagon Devices, USA) on 4 leaves per tree, that were fully expanded and exposed to radiation.

### Leaf and wood traits

The leaf mass per unit area (LMA; g cm^−2^) and wood density (WD; g cm^−3^) were measured in winter 2016, when the growth rate of *P. tamarugo* decreased. Leaf and branch samples were taken from the northeastern side of the trees in <1 and 6–9 m GWDrs (*n* = 5 and *n* = 7, respectively), stored in bags, placed in coolers with gel packs, and taken to the laboratory for analysis. Leaf area was measured from a composed sample of leaf (~50 g of fresh weight per tree) using digital image analysis in ImageJ (Schindelin et al., 2015). The samples were placed in a forced air oven (Venticell, MMM Group) at 60°C until they reached a constant weight to determine leaf dry matter. LMA (g cm^−2^) was estimated as the ratio between the leaf dry mass and the leaf area of each sample. The branches (two per tree) were cut into segments of roughly 10 cm from the base and cut again in six cylinders of similar diameter for wood density measurement. The bark and spines were removed from each segment. Fresh wood volume was determined using the dimensional method (Pérez et al., 2013), measuring the diameter (average from the diameter at the base, center, and end of the segment) and length of the cylinder. Subsequently, the samples were placed in a 60°C oven until constant weight, and WD was estimated as the ratio between the dry mass and fresh volume of each segment.

The color of eight leaves per tree was measured during the January 2016 field campaign. For this, a Munsel® color chart for plant tissue was used in fully expanded leaves exposed to radiation in the northeastern face of the tree, and the frequency of colors in each tree was registered.

### Stable isotope analysis and intrinsic water use efficiency (iWUE)

The observed variation in intrinsic water use efficiency may be due to net assimilation (A_n_) variation (with a constant gs) or to g_s_ changes (with constant A_n_), Scheidegger et al. (2000) proposed a model of analysis based on the use of the different possible associations between δ^13^C and δ^18^O. The use of δ^18^O is based on its negative association with g_s_, when g_s_ is the limiting factor for transpiration (Farquhar et al., 2007). Two assumptions had to be met for g_s_ estimation: the air relative humidity and δ^18^O of the water source should not differ between sites (Farquhar et al., 2007; Roden and Siegwolf, 2012). Our study was conducted in a forest of isolated individuals, and the groups of evaluated trees were less than 4 km apart across a homogeneous topography, so atmospheric conditions were similar between sites. In addition, the groundwater δ^18^O along the studied transect had an average value of −6.2 ± 0.19‰ (unpublished data, personal communication, Geohidrologia Consultores, ARCADIS, Chile), so the assumptions were met.

Although Scheidegger et al. (2000) developed their interpretation model in herbaceous species under natural conditions, it has also been applied to woody species studies such as *Quercus frainetto* Ten. under natural conditions (Colangelo et al., 2017), as well as in retrospective studies in *Fagus sylvatica* and *Nothofagus* spp. (Tognetti et al., 2014), and in Scots pine (Voltas et al., 2013). The efficacy of the model has been evaluated by Roden and Farquhar (2012) measuring δ^13^C and δ^18^O in growth rings of *Eucalyptus globulus* Labill. and *Pinus radiata* D. under controlled conditions. The authors evaluated the effect of different abiotic stresses, (low irradiance, nitrogen deficit, heat, and drought) on the isotopic composition of wood and cellulose simulating climate change. In their assay the authors hypothesized that it was possible to predict A_n_ and g_s_ behavior from the stable isotopes values. Although the dual isotopic model did not perform best in all the evaluated scenarios, it was particularly efficient under drought and heat stress (under low relative humidity and light restriction only).

In order to determine the leaf isotopic composition of ^13^C (δ^13^C; ‰) and ^18^O (δ^18^O; ‰), a sample of leaves was taken from the trees at the GWDrs <1 and 6–9 m in summer, and from all trees along the transect in winter. Sampling was carried out in branches with northeastern exposure at a height of roughly 1.5 m, selecting fully expanded leaves of good phytosanitary and nutritional status. The size of each sample was ~50 g of fresh weight per tree. The leaves were dried at 60°C in a forced air oven (Venticell, MMM Group) until constant weight, and then crushed in a mill to a homogeneous powder. Two subsamples from each sample were weighed with an analytical balance (Precisa 125A) in tin and silver capsules for δ^13^C and δ^18^O measurements respectively. Each leaf sample isotopic composition was determined using standard procedures at the Stable Isotope Laboratory at the Faculty of Agronomic Sciences (University of Chile), with an INTEGRA2 isotopic ratio mass spectrometer (IRMS) (Sercon Ltd. Cheshire, UK), with a precision of 0.3 and 0.5‰ for δ^13^C and δ^18^O, respectively. The measurements were standardized to the relative values of Pee Dee Belemnite (PDB) and Vienna Standard Mean Ocean Water (VSMOW) for carbon and oxygen, respectively.

(1) $$\begin{array}{r} \delta \;\left(‰\right)=\frac{R_{sample}}{R_{standard}}-1 \end{array}$$

where R_sample_ and R_standard_ are the isotopic ratios of ^13^C/^12^C or ^18^O/^16^O of the samples and PDB or VSMOW, respectively. Carbon isotope discrimination (Δ^13^C; ‰) was estimated as follows (Farquhar et al., 1982):

(2) $$\begin{array}{r} \Delta^{13}C=\frac{{\delta^{13}C}_{air}-{\delta^{13}C}_{plant}}{1+{\delta^{13}C}_{plant}} \end{array}$$

where ${\delta^{13}C}_{air}$ is the carbon isotope composition of air, with a value of -8.15‰ (CDIAC, 2008, available at http://cdiac.ess-dive.lbl.gov/trends/co2/iso-sio/iso-sio.html), and ${\delta^{13}C}_{plant}$ (‰) is the carbon isotopic composition of leaf tissue (Equation 2). Given that Δ^13^C is related to the ratio between the concentration of carbon dioxide in the intercellular spaces and the atmosphere (C_i_/C_a_), and assuming that stomatal conductance to water is 1.6 times the conductance to CO_2_, the intrinsic water use efficiency (iWUE) can be estimated from Δ^13^C as follows:

(3) $$\begin{array}{r} iWUE=\left[1-\left(\frac{\Delta^{13}C-a}{b-a}\right)\right]\frac{C_{a}}{1.6} \end{array}$$

where *a* is the fractionation caused by the diffusion of CO_2_ from the atmosphere to the intercellular spaces of the leaf (4.4‰), *b* is the fractionation caused by the isotope discrimination of ^13^CO_2_ of RuBP carboxylase (27‰), and *C*_*a*_ (μmol mol^−1^) is the atmospheric carbon dioxide concentration which was obtained from NOAA ESRL (https://www.esrl.noaa.gov), being 402.52 and 402.25 μmol mol^−1^ in January and August 2016, respectively.

### Tree biometric variables

The green canopy cover fraction (*f*GCC) of trees at the GWDrs <1 and 6–9 m was estimated using digital image analysis based on objects using eCognition® Developer. The variable was defined as the green surface of a side of a *P. tamarugo* tree in the total area of the same side and was determined according to the methodology described by Chávez et al. (2013) and Garrido et al. (2016). The relationship between *f*GCC and the total cross-sectional trunk area of each tree (ΣCSA) was calculated as an approximation to the leaf area: sapwood area (A_l_: A_s_).

In order to assess the magnitude of *f*GCC change within the year, a relative difference of green coverage from January to August (R*f*GCC) was calculated as follows.

(4) $$\begin{array}{r} \text{R}fGCC=\frac{{\text{f}GCC}_{jan}-{\text{f}GCC}_{aug}}{{\text{f}GCC}_{jan}} \end{array}$$

where *f*GCC_*jan*_ and *f*GCC_*aug*_ are the highest and lowest *f*GCC respectively.

The number of trunks per tree and their perimeters were measured at one-meter height with a tape to estimate the cross-sectional area of each trunk (CSA, m^2^). Strong allometric relationships between sapwood area and cross-sectional area have been reported under various conditions (Eamus et al., 2000; Kelley et al., 2007; Zolfaghar et al., 2017). Thus, CSA can be used as an approximation for the sectional area of water conduction tissue. The height of the tree (m) was measured with a Tandem-360R/PC altimeter (Suunto, Finland), and the crown size (m^2^) was estimated as the average circular area between the largest and smallest diameter of each tree. The average internode length (IntLength; cm) was measured in 20 terminal branches per tree at ~1.5 m height in the north-east face and calculated as the ratio between the length of the branch and the number of internodes.

### Statistical analysis

All statistical analyses were done using the InfoStat interface (Di Rienzo et al., 2011) with R version 3.2.1 (R Core Team, 2015). Mixed general linear models with a structure of repeated measurements over time were used, where GWDr, season (winter and summer), and the interaction between GWDr and season were fixed factors. The autocorrelation structure of the repeated measurements on the same individual in summer and winter was modeled using a compound symmetry function (Pinheiro and Bates, 2000). With variables measured only in winter, GWDr was defined as a fixed factor and the tree as a random factor, and a heteroscedastic model was used. Model selection was based on the fulfillment of the statistical analysis assumptions and by using AIC index values (Akaike information criterion; Bozdogan, 1987) as the selection criteria. *Post-hoc* Fischer's LSD analyses were used when appropriate.

## Results

### Environment of the study site and *P. tamarugo* water status

**Figure 2:**
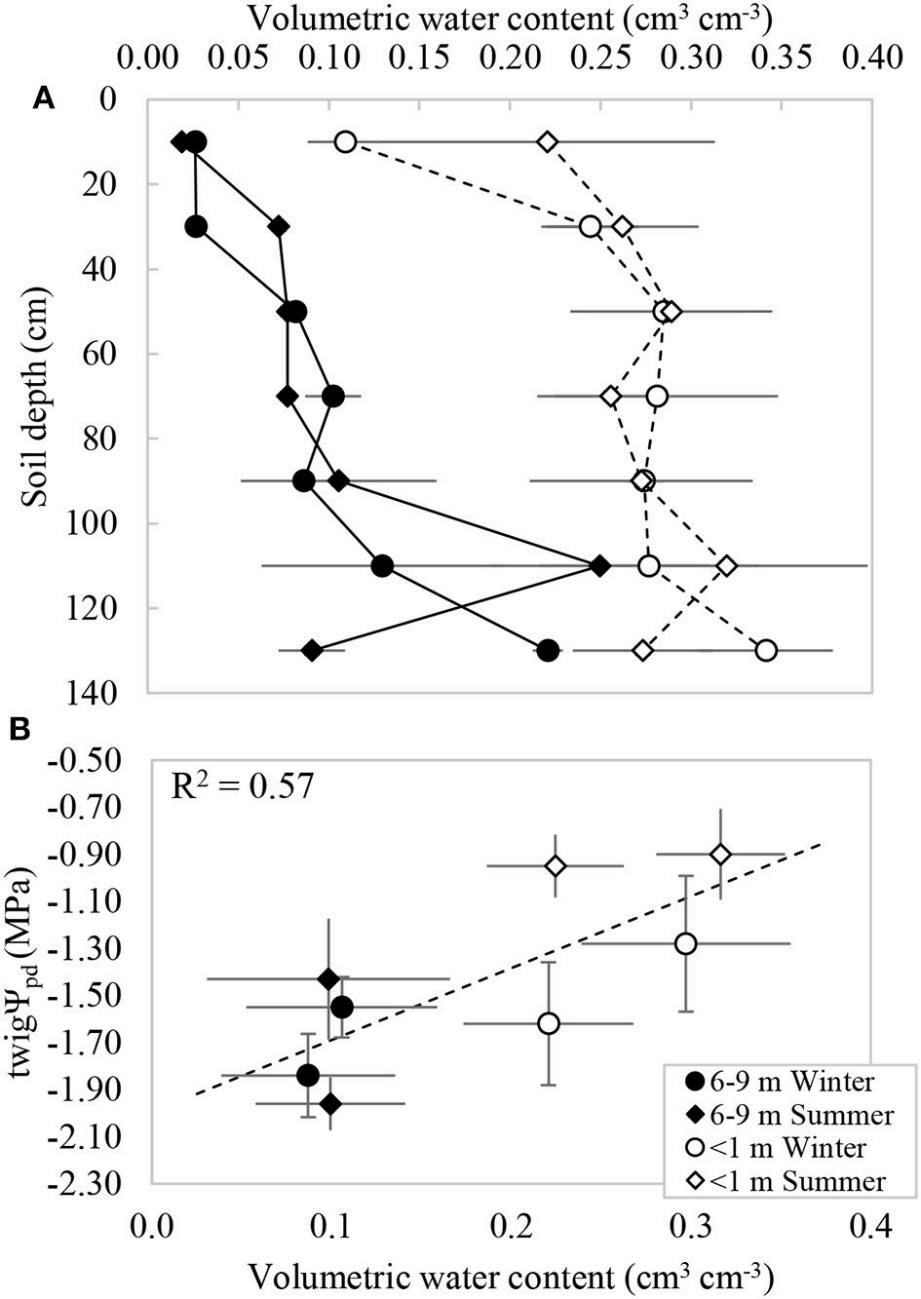
**(A)** Volumetric water content measured under *P. tamarugo* in two groundwater depletion ranges during winter (circles) and summer (diamonds) 2016. **(B)** Relationship between the pre-dawn branch water potential and volumetric water content (*twig*Ψ_pd_ = 3.1VWC −1.99). Vertical and horizontal bars represent ± 1 S.E.

The GWDr × Season interaction was not significant for soil volumetric water content (θ; *p* = 0.808), but GWDr was significant independently (*p* < 0.001). The average soil profile volumetric water content ± standard error (*n* = 4) was 0.265 ± 0.031 cm^3^ cm^−3^ in the GWDr <1 m; roughly 2.5 times higher than that observed in the 6–9 m, which had an average of 0.098 ± 0.035. In both GWDrs water content tended to increase with soil depth (Figure 2A). This was consistent with the presence of roots observed in the field, which increased with depth. The volumetric soil water content at each tree and in each sampling season was positively and significantly correlated to the *twig*Ψ_*pd*_ measured in the same trees (Figure 2B).

The chemical soil analysis (Table 2) from the two test pits in each GWDr indicated that the soil from the <1 m tended to have a similar pH, lower EC and K, and higher OM, N, and P-Olsen contents (on average between 50 and 100 cm deep) compared to the 6–9 m GWDr. In the soil profile, OM, N, K, and P-Olsen tended to be higher in the upper 50 cm than deeper in the soil.

**Table 2:** Mean values ± 1 S.D. (*n* = 2) of pH, electrical conductivity (EC), organic matter content (%), total nitrogen (N), Olsen phosphorous (P), and potassium (K), measured under *P. tamarugo* at 50 and 100 cm depth, at two ranges of groundwater depletion (Salar de Llamara, Pampa del Tamarugal).

	**Groundwater depth range**			
	<**1 m**		**6–9 m**	
Soil depth (cm)	50	100	50	100
pH	8.5 ± 0.00	8.5 ± 0.00	8.6 ± 0.07	8.5 ± 0.00
EC (dS m^−1^)	20.0 ± 3.54	19.9 ± 2.76	40.6 ± 1.06	17.2 ± 3.61
OM (%)	0.9 ± 0.64	0.4 ± 0.00	0.5 ± 0.51	0.1 ± 0.04
N (mg Kg^−1^)	14.5 ± 10.61	7.0 ± 4.24	10.5 ± 2.12	7.5 ± 2.12
P (mg Kg^−1^)	17.0 ± 1.41	5.0 ± 2.83	7.5 ± 0.71	4.5 ± 3.54
K (mg Kg^−1^)	864.5 ± 2.12	659.0 ± 1.41	1126 ± 226.27	526 ± 189.5

### Water stress adjustment of morpho-physiological and structural traits at canopy level

**Figure 3:**
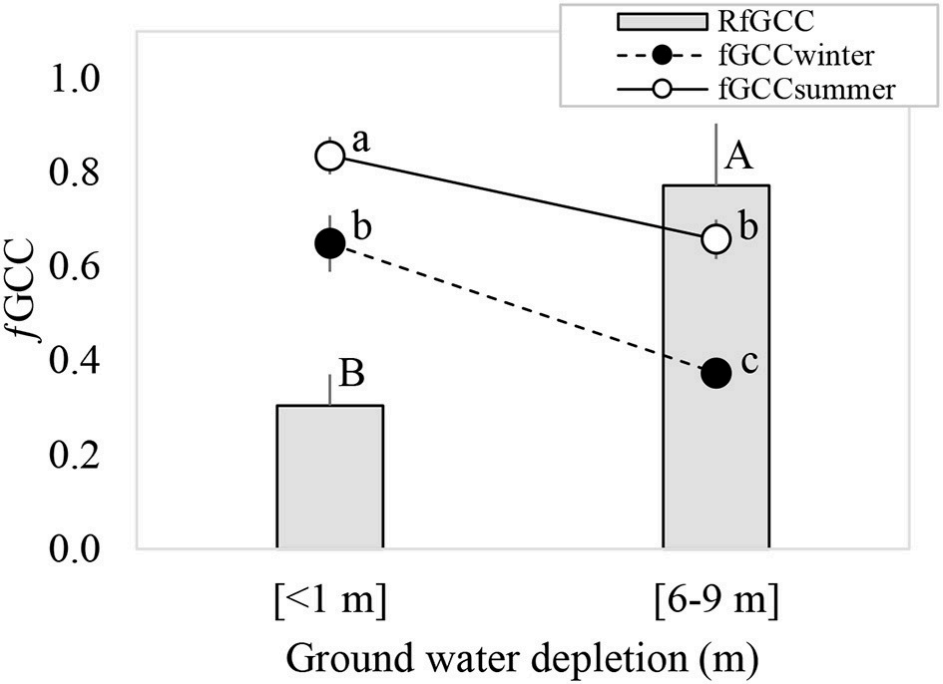
Green canopy cover fraction (*f*GCC) (circles) measured in winter and summer, and relative loss of summer-winter green coverage (R*f*GCC) of *P. tamarugo* under <1 m (*n* = 5) and 6–9 m (*n* = 7) groundwater depletion. Different letters indicate significant differences (*p* < 0.05), according to Fisher's LSD *post-hoc* analysis.

The GWDr × Season interaction was not significant for *f*GCC. The <1 m GWDr had a significantly higher *f*GCC (*p* = 0.0002) than the 6–9 m GWDr, and *f*GCC was higher in summer than winter (*p* = 0.0001). Winter defoliation estimated through R*f*GCC was significantly higher (*p* = 0.0194) in the 6–9 m GWDr (Figure 3).

LMA was significantly higher in the 6–9 m GWDr, having a significantly smaller leaflet area compared to the <1 m GWDr. The individual leaflet mass was not different between GWDrs. Nor there were significant differences in WD (Table 3). Leaf color had a hue (5GY) and chroma (4) equal in each tree measured, but trees in the 6–9 m GWDr had a higher frequency of values 5 of chroma (63%), that is, lighter green respect to <1 m GWDr which had the same proportion of values 5 and 4.

**Table 3:** Leaf mass per unit area (LMA), wood density (WD), individual leaf area (LA), and individual leaf mass (LM) measured in winter 2016 in *P. tamarugo* subjected to <1 (*n* = 5) and 6–9 m (*n* = 7) groundwater depletion ranges (Salar de Llamara, Pampa del Tamarugal).

	**LMA**	**WD**	**LA**	**LM**
**GWD range**	**g cm^−2^**	**g cm^−3^**	**cm^2^**	**g**
[<1 m]	0.011 ± 0.001 B	0.696 ± 0.031 A	0.114 ± 0.008 A	1.21 ± 0.168 A
[6–9 m]	0.015 ± 0.001 A	0.720 ± 0.022 A	0.073 ± 0.004 B	1.05 ± 0.032 A
**SIGNIFICANCE (*****P*****-VALUE)**				
GWD range	0.0141	0.5481	0.0034	0.3991

*Different letters vertically indicate significant differences (p < 0.05) according to Fisher's LSD post-hoc analysis*.

The GWDr×Season interaction was significant for iWUE (Table 4). The GWDr <1 m in winter had the lowest iWUE (75.9 μmol CO_2_ mol H_2_O^−1^). In all other cases, *P. tamarugo* had an iWUE greater than 90 μmol CO_2_ mol H_2_O^−1^. The GWDr × Season interaction was significant for δ^13^C (*p* = 0.0037), δ^13^C being lower in the GWDr <1 m in winter, while for δ^18^O the interaction was not significant. The greater ^18^O enrichment was found in summer, independently in the 6–9m GWDr (Table 4).

**Table 4:** Intrinsic water use efficiency, δ^13^C, and δ^18^O of *Prosopis tamarugo* subjected to <1 m (*n* = 5) and 6–9 m (*n* = 7) of groundwater depletion ranges, in summer and winter of 2016 (Salar de Llamara, Pampa del Tamarugal).

	**iWUE μmol CO_2_ mol H_2_O^−1^**	**δ^13^C ‰**	**δ^18^O ‰**
**GWD RANGE**			
[<1 m]	85.1 ± 2.37	−26.8 ± 0.30	31.2 ± 0.17 B
[6–9 m]	91.4 ± 1.99	−26.3 ± 0.17	32.4 ± 0.35 A
**SEASON**			
Summer	93.2 ± 1.61	−26.1 ± 0.16	32.3 ± 0.36 A
Winter	83.2 ± 1.95	−27.0 ± 0.23	31.3 ± 0.11 B
**INTERACTION**			
[<1 m] x Summer	94.3 ± 2.81 A	−26.0 ± 0.30 A	31.8 ± 0.30 A
[<1 m] x Winter	75.9 ± 2.64 B	−27.7 ± 0.38 B	30.7 ± 0.13 B
[6–9 m] x Summer	92.1 ± 1.56 A	−26.2 ± 0.13 A	32.8 ± 0.66 A
[6–9 m] x Winter	90.6 ± 2.87 A	−26.4 ± 0.26 A	32.0 ± 0.17 A
**SIGNIFICANCE (*****P*****-VALUE)**			
GWD range	0.0102	0.1470	0.0090
Season	0.0001	0.0006	0.0274
GWD range x Season	0.0002	0.0037	0.6359

*Different letters indicate significant differences (p < 0.05) according to Fisher's LSD post-hoc analysis.Values without letters indicate that the interaction was significant and no comparison among means can be done*.

**Figure 4:**
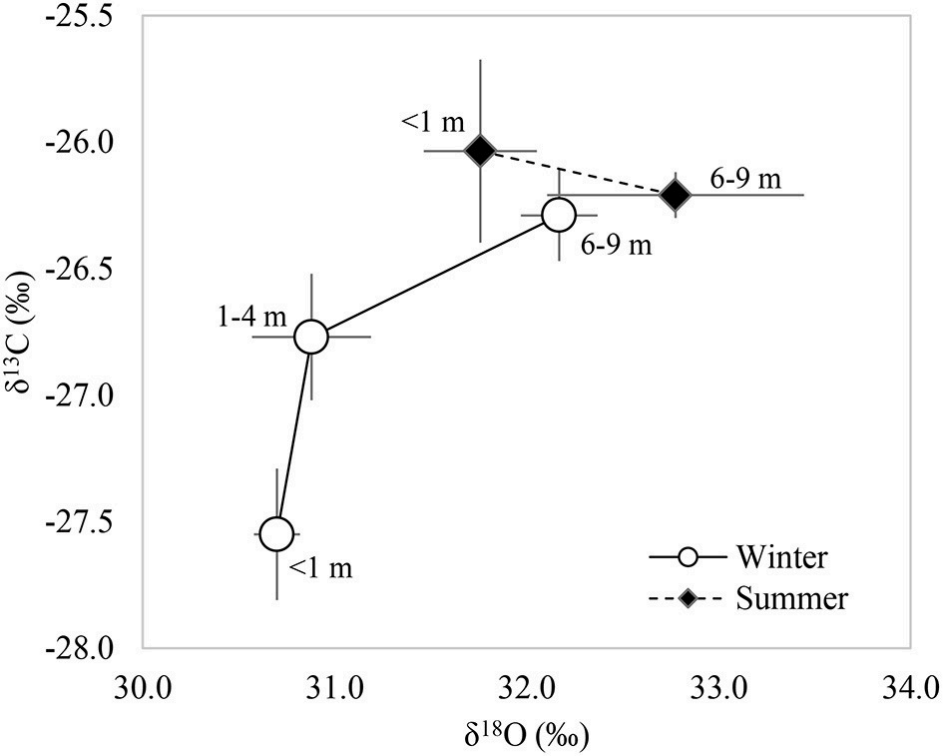
Relationship of the ^13^C and ^18^O foliar isotopic compositions measured in <1 (*n* = 5), 1–4 m (*n* = 4), and 6–9 m (*n* = 7) groundwater depletion ranges in winter and summer 2016.

An increase in the concentration of both ^13^C and ^18^O was observed as the groundwater depletion increased during winter. In contrast, the isotopic composition of the leaves δ^13^C measured in summer was similar among GWDrs, but δ^18^O was higher in areas with groundwater depletion (Figure 4). An increase in δ^18^O with no variation in δ^13^C (as observed during summer) is interpreted as a decrease in stomatal conductance coordinated with a decrease in net assimilation with increasing GWD; while an increase in both δ^13^C and δ^18^O (as observed in winter) is interpreted as a decrease in stomatal conductance, without significant variation in net assimilation (Scheidegger et al., 2000).

**Figure 5:**
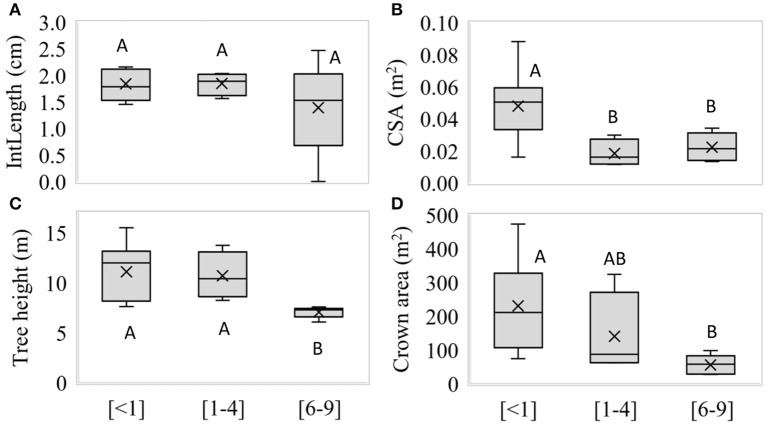
**(A)** length of internodes, **(B)** cross-sectional area of stem, **(C)** tree height, and **(D)** crown size along <1 (*n* = 5), 1–4 m (*n* = 4), and 6–9 m (*n* = 7) groundwater depletion, measured in winter 2016. Different letters indicate significant differences (*p* < 0.05), according to Fisher's LSD *post-hoc* analysis.

No significant differences were observed between GWDr for IntLength (*p* = 0.5503, Figure 5A). The CSA was significantly higher (*p* = 0.0314) in the <1 m, decreasing at greater GWDrs (Figure 5B). The trees in the 6–9 m GWDr were significantly shorter (*p* = 0.0018, Figure 5C). Significant differences in crown size (*p* = 0.0195) were observed, which increased at lower GWDrs (Figure 5D).

### *P. tamarugo* water relationships

The defoliation and restriction of radial trunk growth maintained a constant *f*GCC CSA^−1^ relation among GWDrs. The GWDr × Season interaction was significant for *twig*Ψ_pd_ (*p* = 0.0009) and *twig*Ψ_md_ (*p* = 0.00389). The largest *twig*Ψ_pd_ was measured at <1 m in summer, while the remaining GWDr × Season interaction was not significant. The largest *twig*Ψ_md_ was observed at the GWDr <1 m during winter, and the smallest was in the 6–9 m in summer. No significant effects of GWDr, season, or their interaction on g_s_ were observed (Table 5). The differences observed between pre-dawn and midday *twig*Ψ between GWDr were consistently larger in summer than winter, 2 and 1 MPa, respectively.

**Table 5:** Relationship between the leaf cover fraction and trunk transverse section area (*f*GCC CSA^−1^), twig water potential at pre-dawn (*twig*Ψpd) and midday (*twig*Ψmd), and stomatal conductance at midday (porometer gs) of *P. tamarugo* subjected to <1 m (*n* = 5) and 6–9 m (*n* = 7) of groundwater depletion ranges during the summer and winter 2016 (Salar de Llamara, Pampa del Tamarugal).

	****f*GCC*** CSA**^−1^ –**	***twigΨ_pd_* Mpa**	***twigΨ_md_* Mpa**	***g_s_* mmol m^−2^ s^−1^**
**GWD RANGE**				
<1 m	2.1 ± 0.68 A	−1.2 ± 0.10	−2.7 ± 0.14	0.108 ± 0.01 A
6–9 m	2.6 ± 0.38 A	−1.8 ± 0.11	−3.1 ± 0.08	0.116 ± 0.01 A
**SEASON**				
Summer	2.9 ± 0.66 A	−1.33 ± 0.09	−3.2 ± 0.10	0.113 ± 0.01 A
Winter	1.8 ± 0.42 A	−1.64 ± 0.09	−2.6 ± 0.08	0.111 ± 0.01 A
**INTERACTION**				
<1 m x Summer	2.4 ± 1.13 A	−0.9 ± 0.05 A	−2.9 ± 0.17 B	0.120 ± 0.01 A
<1 m x Winter	1.8 ± 0.76 A	−1.6 ± 0.16 B	−2.5 ± 0.14 A	0.095 ± 0.01 A
6–9 m x Summer	3.3 ± 0.67 A	−1.8 ± 0.17 B	−3.5 ± 0.10 C	0.105 ± 0.01 A
6–9 m x Winter	1.9 ± 0.36 A	−1.7 ± 0.08 B	−2.6 ± 0.06 AB	0.128 ± 0.02 A
**SIGNIFICANCE (*****P*****-VALUE)**				
GWDr	0.5256	0.0045	0.0551	0.5334
Season	0.2101	0.0056	<0.0001	0.9149
GWDr x Season	0.6263	0.0009	0.0038	0.0603

*Different letters indicate significant differences (p < 0.05) according to Fisher's LSD post-hoc analysis. Values without letters indicate that the interaction was significant and no comparison among means can be done*.

## Discussion

### Acclimation of *P. tamarugo* under long-term groundwater depletion

Using Darcy's Law (Equation 5) we can evaluate restrictions and mechanisms of homeostatic compensation on stomatal conductance (g_s_) at canopy level (McDowell and Allen, 2015) as follows:

(5) $$\begin{array}{r} g_{s}=\frac{A_{s}k_{s}\left({\text{Ψ}}_{s}-{\text{Ψ}}_{l}\right)}{h_{w}\eta A_{l}D_{s}} \end{array}$$

where A_s_ is the conductive area (m^2^), k_s_ is the specific hydraulic conductivity (m s^−1^), Ψ_s_-Ψ_l_ is the soil-leaf water potential difference (ΔΨ; MPa), *h*_*w*_ is the tree height (m) (an approximation to the hydraulic path length), η is the water viscosity (Pa s), A_l_ is the leaf area (m^2^), and D_s_ is the leaf-to-air vapor pressure deficit (kPa).

In summer and winter, *P. tamarugo* had a ΔΨ across GWDrs of one and two Mpa, respectively. In this context, the smaller cross-sectional area of trees of the groundwater depletion zones (Figure 5; CSA as a proxy of A_s_) would promote a decrease in g_s_ (Equation 5). *P. tamarugo* compensates for this effect restricting growth in height (Figure 5) and having lower green canopy fraction (i.e., a relatively constant *f*GCC CSA^−1^). Keeping wood density constant between GWDrs, it is probable that the maximum specific conductivity of its branches is also maintained constant (Bucci et al., 2004). Even though our study showed no g_s_ differences among GWDrs (Table 5), it is important to keep in mind that this variable is highly dynamic in the field, and it is difficult to achieve adequate representativeness in large canopies (Farquhar et al., 2007).

Previous studies have shown that *P. tamarugo* responds to decreases in water table depth by decreasing the radial growth of branches (Decuyper et al., 2016) and through partial crown defoliation (Ortiz, 2010; Chávez et al., 2013; Garrido et al., 2016). Our study showed that drought induced defoliation occurred along with a leaflet size reduction. The leaves of the remnant canopy of the trees growing at 6–9 m GWDr had higher LMA values. There was a significant leaflet area decreases of 36%, while the leaflet mass decreased by 13% only (non-significant). A higher LMA usually occurs under a reduced leaf area and is associated with tissue that is more resistant to desiccation under water deficit conditions because of a reduction of the boundary layer that helps to maintain a more favorable leaf temperature (Poorter et al., 2009) and a negative association with leaf turgor loss point (Bucci et al., 2004).

The non-increase of cavitation resistance estimated in our study through wood density (Jacobsen et al., 2005, 2007; McCulloh et al., 2012) may be related to the observed winter defoliation increase in the 6–9 m GWDr. A combined effect of water stress and freeze/thaw-induced cavitation (Niu et al., 2017) in an environment with high frost frequency between May and August (minimum of −4.3°C in 2016) would affect water flow to the leaves, promoting senescence. Carevic et al. (2017) observed a decrease in twig hydraulic conductivity of *Prosopis burkartii* during the winter in the Pampa del Tamarugal.

*P. tamarugo* has a dual root system (Sudzuki, 1969) through which it performs “hydraulic lifting.” This process was described by Richards and Caldwell (1987) and confirmed and renamed “hydraulic redistribution” by Burgess et al. (1998). Aravena and Acevedo (1985) found that the ^18^O and ^2^H isotope compositions of the groundwater table and the upper soil water in a tamarugo plantation were similar. Leaves and rootlets tend to be functionally related; both are ephemeral organs, and their main function is the acquisition of resources (Eissenstat et al., 2000), so there would be an association of the foliage and rootlet production (or death) along a resource availability gradient (Hendricks et al., 2006). Thus, the lower presence of roots observed in the field (visual observations only) and lower humidity in the upper soil under *P. tamaurugo* at 6–9 m GWDr could have a common origin with defoliation.

### Analysis of the intrinsic water use efficiency of *P. tamarugo* using stable isotopes in the leaf tissue

Intrinsic water use efficiency, defined as the relationship between net photosynthesis and stomatal conductance (A_n_ ${\text{g}}_{\text{s}}^{-1}$; iWUE), integrated over time, and δ^13^C are positively associated (Farquhar et al., 1982). δ^13^C has been used in the study of iWUE of eucalyptus forests subjected to water table depth gradients (Zolfaghar et al., 2017), and in estimating root depth and groundwater use in different forest communities (Rumman et al., 2017).

In summer, leaves showed a discrete δ^18^O increase (Figure 4) but no changes in δ^13^C with increasing GWDr, which would indicate that GWDr lowering has no effect on iWUE due to a coordinated decrease in both gs and An over time, according to the dual isotopic model (Scheidegger et al., 2000). The estimated iWUE for this period was 94.3 and 92.1 μmol CO_2_ mol H_2_O^−1^ in <1 and 6–9 m GWDrs, respectively. During winter, however, (i.e., after the end of the *P. tamarugo* more active growth cycle), the estimated iWUE was 75.9 and 90.6 μmol CO_2_ mol H_2_O^−1^ for <1 and 6–9 m GWDrs, respectively. The greater iWUE at higher groundwater depletion can be the result of a decrease in g_s_ without substantive changes in A_n_ over time (Figure 4), given the positive association between δ^13^C and δ^18^O. This behavior could be explained by the slight effect of groundwater depletion on g_s_ in the remaining *P. tamarugo* canopy, which is not detected through porometry due to its high variability. Following a defoliation event, some species experience a transient increase in the photosynthetic rate as a compensation mechanism against photosynthetic tissue reduction (Pinkard et al., 2007; Eyles et al., 2009). Chávez et al. (2013) observed an increase in carotenoid and chlorophyll a + b concentrations in *P. tamarugo* leaves subjected to prolonged periods of groundwater depletion, that could be due to a concentration phenomenon given LMA increase observed in our study, which could be associated to the maintenance of the net photosynthesis rate at deeper groundwater levels.

### Ecological implications of groundwater depletion in pampa del tamarugal ecosystem

Among the different ecological aspects affected by the groundwater depletion in the Pampa del Tamarugal, it has been shown that hydraulic redistribution, i.e., the movement of water from the water table to the upper soil through the root system, has implications at the plant, community, and ecosystem levels (Prieto et al., 2012). Regarding *P. tamarugo*, it could have significant importance in avoidance of root cavitation during the day, because of an increase of water availability during night (Domec et al., 2004), affecting the processes regulated by soil moisture, such as nutrient absorption, nitrogen mineralization, soil respiration, enzymatic activity, and soil productivity determination (Burgess et al., 1998; Cardon et al., 2013). In addition, the effect of hydraulic redistribution as a buffer for the effects of severe soil drying for rhizosphere fungi, has been observed in California Oak savanna during the Mediterranean summer dry period (Querejeta et al., 2007). Specifically, in desert conditions, the reduction of water availability and high salinity affects negatively native *Rhizobium* bacteria, decreasing their population, their inoculation capacity, and symbiotic N_2_ fixation in woody species of the genus *Prosopis* (Zahran, 1999). In our study, the observed higher volumetric water content, OM, N, and P-Olsen, and a lower EC in a condition without groundwater depletion is related to the process affected by hydraulic redistribution mentioned above. In the same way, the observed higher frequency of lighter green leaves under groundwater depletion may be related with lower chlorophyll contents by N deficit. Therefore, in addition to water stress induced by groundwater depletion, availability and symbiotic fixation of N could be affected through the reduction of hydraulic redistribution and increase of the concentration of salts in the upper soil layers, limiting ecosystem sustainability.

To date, important progress has been made in conserving *P. tamarugo*. With the species declared as endangered by the Chilean Ministry of the Environment, the relict forest of the Salar de Llamara became part of the Pampa del Tamarugal National Reserve and water rights ceased to be issued in the area. While these efforts move in the right direction, ensuring the water provision to *P. tamarugo* is a key issue, and it is therefore necessary to reduce the water extraction rate, through water rights expropriation and/or the use of alternative water sources for urban and mining uses, such as desalted seawater. It is also advisable to explore ways that allow the forest to increase its tolerance to water deficit and promote its capacity for recovery, as well as bringing the community and stakeholders closer to the reality of this ecosystem, and the benefits associated with its conservation.

## Conclusions

The morpho-physiological and structural traits adjustment experienced by *P. tamarugo* after 10 years of exposure to groundwater depletion, have allowed it to acclimate and survive. The strategy of *P. tamarugo* consists mainly in an active control of its canopy, maintaining functional but decreased canopy, with small leaves having high stomatal conductance and photosynthesis (estimated from leaf isotopic composition), but exposed to low water potentials. The groundwater depletion negatively affects the root hydraulic redistribution, reducing water availability in the soil under *P. tamarugo* canopy, with known consequences in both, edaphic microbiota, such as atmospheric nitrogen fixation, adding stressors associated to plant nutrition and soil salinity. Although conservation initiatives of the species are moving in the right direction, forest deterioration is expected continue as groundwater depth increases.

## Author contribution

MG designed the experiment, collected data, and drafted this manuscript. HS designed the experiment, collected data, and edited the manuscript. NF edited the manuscript. JA provided logistic support and edited the manuscript. EA followed and discussed upon data collection, provided support through the Soil-Water-Plant Relations Laboratory and edited the manuscript.

### Conflict of interest statement

The authors declare that the research was conducted in the absence of any commercial or financial relationships that could be construed as a potential conflict of interest.
